# Neuroleptic Malignant Syndrome in an Institutionalized Geriatric Patient Following Antipsychotic Switch to Quetiapine: A Fatal Outcome

**DOI:** 10.7759/cureus.89077

**Published:** 2025-07-30

**Authors:** Idalberto Luis Fernandez Eng, Adniel García Cruz, Elizabeth Blanco Espinosa, Susana Fernandez, Marisleydis Perez Santiago

**Affiliations:** 1 Emergency, Hospital Universitario de la Ribera, Valencia, ESP; 2 Geriatrics, Hospital Universitario Arnau de Vilanova, Lleida, ESP; 3 Surgery, Hospital Provincial Universitario “Arnaldo Milián Castro”, Santa Clara, CUB; 4 Critical Care, Universidad de Ciencias Médicas de La Habana, Havana, CUB; 5 General Practice, Universidad de Ciencias Médicas de Camagüey "Carlos J. Finlay", Camagüey, CUB

**Keywords:** emergency medicine, geriatrics, hyperthermia, neuroleptic malignant syndrome, polypharmacy, quetiapine

## Abstract

Neuroleptic malignant syndrome (NMS) is a rare but potentially fatal condition characterized by hyperthermia, severe muscle rigidity, altered mental status, and autonomic dysfunction, associated with dopamine D₂-receptor antagonists. We present the case of a 76-year-old institutionalized male with multiple comorbidities who developed NMS four days after initiating quetiapine, following recent discontinuation of risperidone. The clinical picture included hyperthermia, generalized rigidity, trismus, disorientation, autonomic instability, leukocytosis, hypernatremia, and markedly elevated creatine kinase. Although serotonin syndrome was considered due to concurrent duloxetine use, it was clinically excluded. Despite early treatment with dantrolene and bromocriptine, the patient’s condition deteriorated rapidly, culminating in renal failure, cardiovascular collapse, and death. This case underscores the diagnostic complexity and high mortality risk of NMS in elderly patients with polypharmacy, emphasizing the need for early recognition and prompt management to improve clinical outcomes.

## Introduction

NMS is a rare but potentially fatal idiosyncratic reaction to dopamine D₂-receptor antagonists, most associated with antipsychotic medications [1]. Clinically, it is characterized by a tetrad of hyperthermia, generalized muscle rigidity, altered mental status, and autonomic instability, often accompanied by elevated creatine kinase and leukocytosis. Without prompt recognition and treatment, NMS may progress to rhabdomyolysis, multiorgan failure, and death [2].

Although NMS occurs in only 1% to 1.5% of patients treated with neuroleptics, it carries a high mortality rate once established. Reported mortality ranges from 11% to 38%, primarily due to complications such as cardiac failure, the leading cause of death in these patients [1]. While more recent studies suggest an overall mortality between 5% and 11%, this figure increases significantly in elderly patients with multiple comorbidities and delayed diagnosis. In a large U.S. nationwide inpatient sample (2002-2011), the in-hospital mortality rate for NMS was 5.6%, rising substantially in patients who developed acute kidney injury or respiratory failure [3].

Linked initially to high-potency first-generation antipsychotics, atypical agents such as quetiapine have also been implicated, even at standard or low doses. Vulnerable populations, including elderly patients, individuals with intellectual disabilities, and those with multiple medical comorbidities, are at increased risk [4]. The underlying pathophysiology involves abrupt central dopaminergic blockade, particularly in the hypothalamus and basal ganglia, leading to dysregulation of thermoregulation, motor control, and autonomic function [1].

The risk of neurotoxicity may be further exacerbated by concomitant use of other psychotropic medications, such as serotonin-norepinephrine reuptake inhibitors (SNRIs, e.g., duloxetine), which may interact with antipsychotics both pharmacodynamically and pharmacokinetically. Animal studies suggest that quetiapine may increase plasma levels of duloxetine, potentially enhancing monoaminergic activity and destabilizing autonomic control [5].

Furthermore, age itself has been identified as an independent risk factor, with each additional year increasing the odds of death by approximately 3%. In frail, institutionalized older adults, particularly those with cardiac disease, functional dependence, and polypharmacy, the estimated mortality may approach or exceed 30% [6]. This case underscores the critical importance of thorough medication reconciliation and vigilant clinical monitoring when initiating antipsychotic therapy in medically complex patients, particularly in the setting of polypharmacy and concurrent use of serotonergic agents.

## Case presentation

A 76-year-old institutionalized man with a history of chronic heart failure with reduced ejection fraction, type 2 diabetes mellitus, hypertension, dyslipidemia, untreated obstructive sleep apnea, psoriasis, borderline personality disorder, and mild intellectual disability (Global Deterioration Scale stage 2), indicating very mild cognitive decline [7], presented to the emergency department with fever, tachycardia, tremors, and diaphoresis. Functional status was assessed using the Barthel Index, a validated tool that measures independence in 10 basic activities of daily living, with scores ranging from 0 (complete dependence) to 100 (full independence) [8]. In this case, the patient had a score of 30/100, consistent with severe functional dependence (Table 1). This marked functional decline occurred following a SARS-CoV-2 infection three years earlier, which ultimately led to his institutionalization.

**Table 1:** Barthel index (bolded scores reflect the patient's functional status) Table adapted from the Barthel Index criteria described by Mahoney and Barthel (1965) [8].

Activity	0 points	5 points	10 points	15 points	Patient's score
Feeding	Unable	Needs help	Independent	-	5
Bathing	Dependent	-	Independent	-	0
Grooming	Dependent	-	Independent	-	0
Dressing	Dependent	Needs some help	Independent	-	5
Bowel control	Incontinent	Occasional accident	Continent	-	5
Bladder control	Incontinent	Occasional accident	Continent	-	5
Toilet use	Dependent	Needs some help	Independent	-	0
Transfers (bed to chair and back)	Unable	Major help	Minor help	Independent	5
Mobility (on level surfaces)	Immobile	Wheelchair or assistance	Independent	Walks independently ≥50 meters	5
Stairs	Unable	Needs help	Independent	-	0
Total score					30/100

He was on multiple chronic medications. His symptoms began four days after the initiation of quetiapine at his nursing home, which followed the discontinuation of risperidone five days earlier. His chronic medications included trazodone 50 mg daily, duloxetine (Xeristar) 30 mg daily, gabapentin 300 mg every eight hours, lormetazepam 2 mg at bedtime, and diazepam 5 mg daily, all of which can influence central dopaminergic, serotonergic, or GABAergic pathways and may have contributed to his clinical presentation.

**Figure 1:**
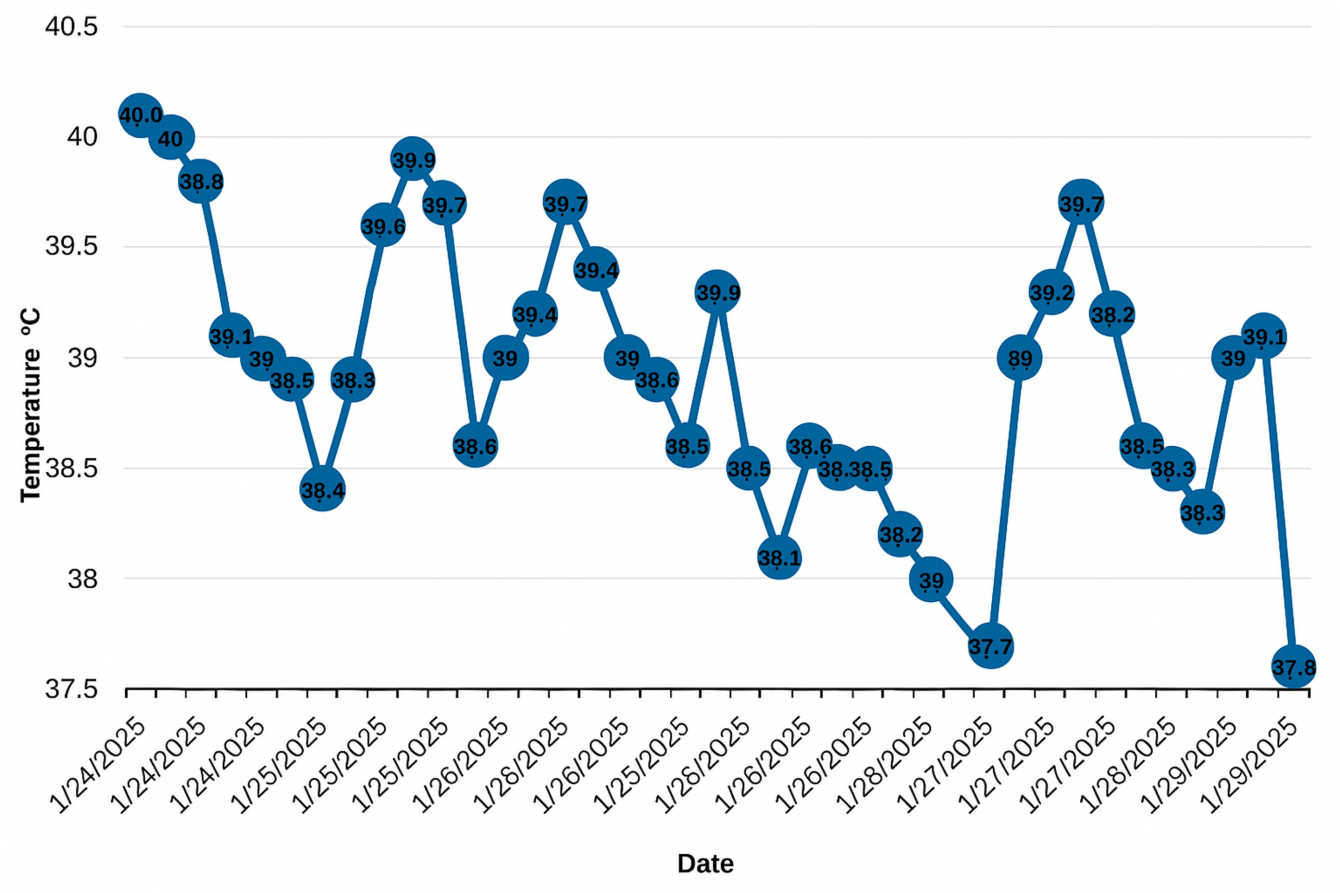
Trend of body temperature over time. The vertical axis represents temperature in degrees Celsius (°C), and the horizontal axis shows the corresponding dates. The graph demonstrates daily fluctuations with multiple recordings per day

On arrival, the patient was alert but disoriented, with a body temperature ranging from 38°C to 40°C, refractory to intravenous paracetamol and metamizole (Figure 1). He exhibited sinus tachycardia (120 bpm), blood pressure of 152/79 mmHg, and oxygen saturation of 94% on room air. Physical examination revealed generalized muscle rigidity, particularly in the upper limbs, a resting tremor, jaw saccadic movements with trismus, and profuse diaphoresis. No focal signs of infection were noted. Laboratory evaluation showed leukocytosis with neutrophilia, hypernatremia, hypokalemia, and elevated creatine kinase, with mildly elevated inflammatory markers (Table 2). Chest and abdominal radiographs were unremarkable, and a brain CT scan showed chronic ischemic changes without acute pathology.

**Table 2:** Laboratory findings on admission to the emergency department GFR (CKD-EPI): glomerular filtration rate estimated by CKD-EPI equation, AST: aspartate aminotransferase, ALT: alanine aminotransferase, GGT: gamma-glutamyl transferase, MCV: mean corpuscular volume, MCH: mean corpuscular hemoglobin, RDW: red cell distribution width, INR: international normalized ratio, aPTT: activated partial thromboplastin time, CRP: C-reactive protein, RBCs: red blood cells, WBCs: white blood cells, MPV: mean platelet volume

Test	Result	Reference range
Glucose	136 mg/dL	74-100 mg/dL
Creatinine	1.19 mg/dL	0.67-1.17 mg/dl
Urea	46 mg/dL	19-62 mg/dL
GFR (CKD-EPI)	63 mL/min/1.73m^2^	-
AST (GOT)	22 U/L	3-50 U/L
ALT (GPT)	10 U/L	3-50 U/L
Alkaline phosphatase	31 U/L	40-130 U/L
GGT	10 U/L	4-55 U/L
Total bilirubin	0.66 mg/dL	0.3-1.2 mg/dL
Direct bilirubin	–	0-0.3
Indirect bilirubin	Not calculable	0-1
Amylase	116 U/L	28-100 U/L
Total proteins	7.34 g/dL	6.60-8.30 g/dL
Albumin	3.4 g/dL	3.2-4.8 g/dL
Sodium	147 mmol/L	136-145 mmol/L
Potassium	3.49 mmol/L	3.6-5.2 mmol/L
Calcium	6.46 mg/dL	8.80-10.6 mg/dL
Chloride	98 mmol/L	101-109 mmol/L
CRP	5.5 mg/L	0.2-5.0 mg/L
Creatine kinase	279 U/L	45-196 U/L
Procalcitonin	0.04 ng/ml	-
pH	7.44	7.33-7.42
pCO₂	45 mmHg	38-50 mmHg
pO₂	52 mmHg	30-50 mmHg
HCO₃⁻	30.6 mmol/L	23-27 mmol/L
O₂ saturation	83.6%	60-85%
Base excess	5.6 mmol/L	(-)2.0 mmol/L
Lactate	2.2 mmol/L	0.5-2.0 mmol/L
RBCs	4.41 x10¹²/L	4.5-6.1 x10¹²/L
Hemoglobin	13.7 g/dL	13-18 g/dL
Hematocrit	42.2%	38-52%
MCV	95.7 fL	80-100 fL
MCH	31.1 pg	26-34 pg
MCHC	32.5 g/dL	32-36 g/dL
RDW	13.1%	11.5-14.5%
WBCs	13.07 x10⁹/L	4.8-10.8 x10⁹/L
Neutrophils	76.6%	40-75%
Lymphocytes	14.5%	17-51%
Monocytes	7%	1.7-10%
Eosinophils	1.1%	0.1-7%
Basophils	0.8%	0-1.2%
Platelets	317 x10⁹/L	140-450 x10⁹/L
MPV	11 fL	6.8-12.5 fL
Prothrombin time (ratio)	1.2	0.86-1.26
INR	1.21	0.9-1.2
aPTT	26.4 sec	24-40 sec
Fibrinogen	5.7 g/L	2.8-4.7 g/L

The diagnosis of NMS was established based on DSM-5 criteria (Table 3). Intravenous dantrolene (2 mg/kg) was administered, resulting in partial improvement of muscle rigidity, although hyperthermia persisted. Maintenance doses of 1 mg/kg every six hours were continued due to ongoing clinical symptoms. A comprehensive infectious workup, including blood and urine cultures and other laboratory and imaging studies, returned negative results. Serotonin syndrome (SS) was considered in the differential diagnosis due to concurrent treatment with duloxetine, an SNRI. However, it was clinically excluded due to the absence of characteristic findings such as gastrointestinal symptoms, clonus, and hyperreflexia.

**Table 3:** DSM-5 diagnostic criteria for NMS According to the DSM-5, diagnosis requires the presence of criteria A, B, and C, in addition to two or more of the remaining criteria. Moreover, criterion G, the exclusion of other medical or neurological conditions, is essential for establishing an accurate diagnosis. Diagnostic structure and content adapted from Simon et al. (2023) [6]. CK: creatine kinase, DSM-5: Diagnostic and Statistical Manual of Mental Disorders, Fifth Edition, NMS: neuroleptic malignant syndrome

Criterion	Description
A. Exposure to a dopamine antagonist	Recent use of neuroleptic (antipsychotic) medication or a dopamine antagonist
B. Severe muscle rigidity	Marked generalized muscular rigidity
C. Fever	Elevated body temperature (usually >38°C / 100.4°F)
D. Autonomic instability	Symptoms such as tachycardia, labile or elevated blood pressure, diaphoresis, or tachypnea
E. Altered mental status	Changes in consciousness range from agitation to coma
F. Laboratory abnormalities	Elevated CK, leukocytosis, metabolic acidosis
G. Exclusion of other causes	Symptoms not better explained by other medical or neurological conditions

Furthermore, SS typically presents with less severe hyperthermia and rigidity. Malignant hyperthermia (MH) was also ruled out, as there was no prior exposure to triggering agents such as depolarizing neuromuscular blockers (e.g., succinylcholine). A comparative summary of clinical features among NMS, SS, and MH is shown in Table 4, based on established diagnostic criteria [1-3,6].

**Table 4:** Comparison of NMS, SS, and MH Diagnostic features and clinical distinctions adapted from Sweileh et al. (2024) [1], Schönfeldt-Lecuona et al. (2020) [2], Pelonero et al. (1998) [3], and Simon et al. (2023) [6]. CK: creatine kinase, SSRIs: selective serotonin reuptake inhibitors, SNRIs: serotonin-norepinephrine reuptake inhibitors, NMS: neuroleptic malignant syndrome, SS: serotonin syndrome, MH: malignant hyperthermia

Characteristic	NMS	SS	MH
Causative agent	Antipsychotics (dopamine antagonists)	Serotonergic agents (SSRIs, SNRIs, etc.)	Inhaled anesthetics, succinylcholine
Onset	Hours to days after initiation or dosage change	Hours after initiation or dose increase	Minutes to hours after anesthesia
Body temperature	>38°C, may be very high	Moderate to high	Very high, may exceed 41°C
Muscle rigidity	Generalized, "cogwheel" rigidity	Myoclonus, no severe rigidity	Severe muscle rigidity
Neurological changes	Confusion, stupor, coma	Agitation, hyperreflexia, clonus	Hypoxia, coma in advanced stages
Autonomic signs	Labile blood pressure, tachycardia	Tachycardia, diaphoresis	Tachycardia, hypertension
Laboratory findings	Markedly elevated CK, leukocytosis	Moderately elevated CK	Markedly elevated CK
Treatment	Discontinue antipsychotic, dantrolene, bromocriptine	Discontinue serotonergic agents, benzodiazepines, cyproheptadine	Dantrolene, supportive care

**Figure 2:**
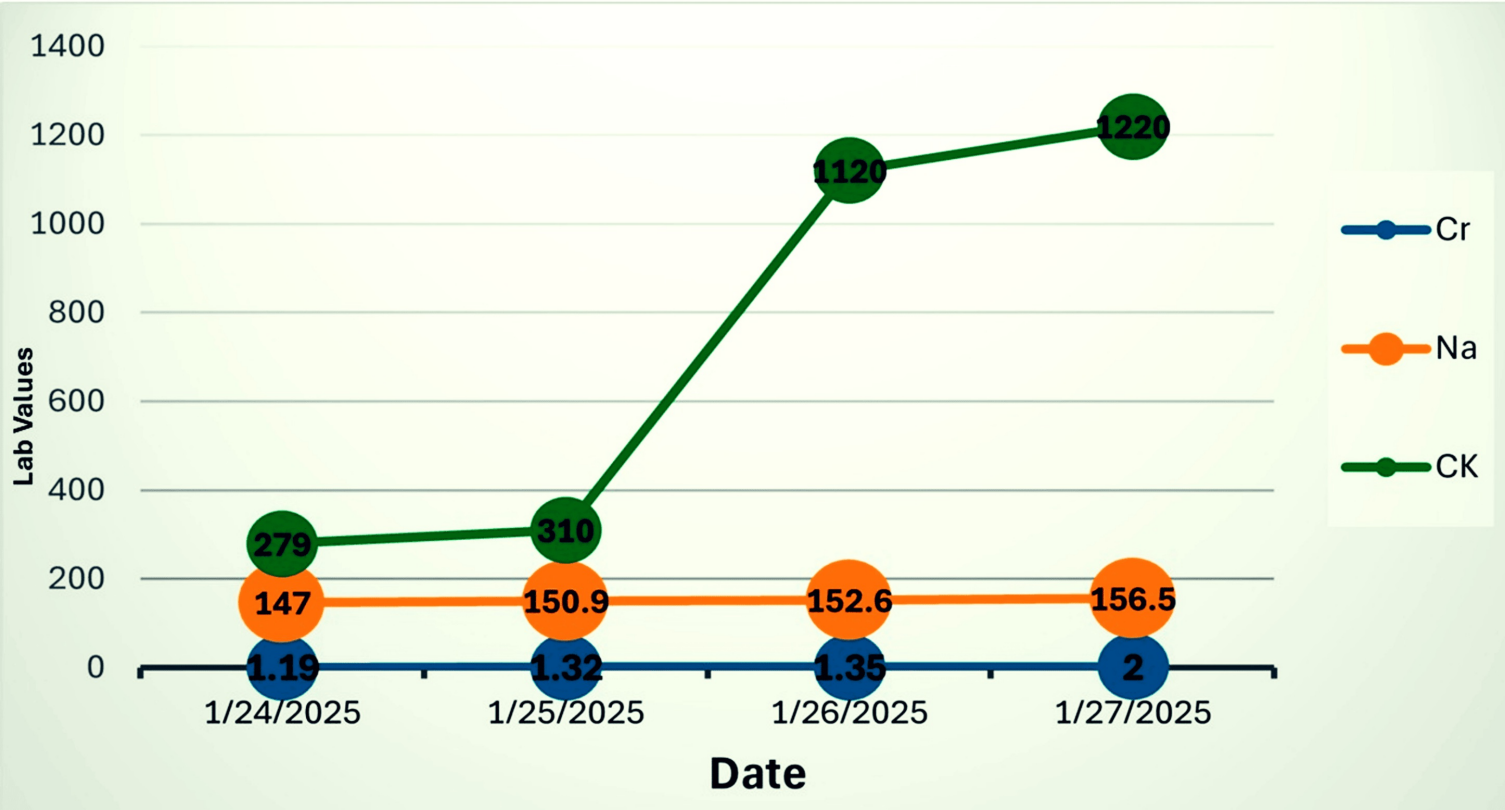
Evolution of serum Cr, Na, and CK levels over several consecutive days Cr: creatinine (mg/dL), Na: sodium (mmol/L), CK: creatine kinase (U/L)

The patient was admitted for close monitoring and supportive care. Bromocriptine (2.5 mg every six hours) was added via nasogastric tube during the hospital admission. Over the next five days, his clinical status progressively deteriorated. He developed worsening mental status, rising hypernatremia (158 mmol/L), hypocalcemia, and progressive renal dysfunction (Figure 2). Although renal replacement therapy was considered, it was ultimately not initiated due to the patient’s poor baseline functional status and overall unfavorable prognosis. Despite intensive care and correction of fluid and electrolyte imbalances, he developed hypoxia and hemodynamic instability, culminating in a cardiac arrest that was unresponsive to advanced resuscitation efforts.

The severe hypernatremia posed a significant clinical challenge, as it can worsen neurological status and increase mortality risk. Managing such electrolyte disturbances requires careful fluid balance and timely correction, which was complicated by the patient’s renal dysfunction and hemodynamic instability. Furthermore, his multiple comorbidities and frailty limited his physiological reserve, reducing responsiveness to supportive measures. This case underscores the complexity of managing systemic complications in NMS. It highlights the critical need for vigilant monitoring and aggressive metabolic management, although outcomes may remain poor in critically ill patients.

## Discussion

Several poor prognostic factors, including advanced age, heart failure, severe functional dependence, and polypharmacy, likely influenced the patient's outcome in this case. Despite prompt treatment with dantrolene and bromocriptine, he developed multiorgan dysfunction, including worsening renal failure, profound hypernatremia, and, ultimately, cardiac arrest, a common terminal event in fatal NMS due to autonomic instability. This constellation of factors is consistent with the elevated mortality risk described in elderly patients with complex medical profiles [6].

While NMS has long been associated with first-generation antipsychotics, it has also been reported with atypical second-generation agents and various other drug classes [9]. Although NMS can affect individuals across all age groups, most reported cases occur in young adults. Age itself is not considered a direct risk factor for the onset of NMS, though older adults tend to have worse outcomes. A trend toward male predominance has been noted in several series; however, this likely reflects patterns of antipsychotic use rather than true biological susceptibility [10].

A concerning trend in clinical practice is the increasing use of psychiatric polypharmacy, including the co-prescription of antidepressants and antipsychotics, sometimes in the absence of clear diagnostic indications. This phenomenon is widespread in institutionalized individuals, those with high dependency levels, or intellectual disabilities [1].

Numerous medications, including antipsychotics and antiemetics, have been implicated in the development of NMS. A unifying mechanism among these agents is central dopaminergic blockade. The reaction is not allergic but idiosyncratic, and the precise pathophysiology remains poorly understood. Recognized risk factors include high doses, rapid dose escalation, parenteral administration, abrupt switching of dopamine antagonists, and dehydration, all of which increase the likelihood of triggering the syndrome. NMS has also been observed following withdrawal of dopaminergic medications such as levodopa [10].

In this case, the patient developed symptoms four days after initiating low-dose quetiapine (25 mg twice daily) and five days after discontinuing risperidone. The diagnosis of NMS was established based on DSM-5 criteria (Table 2), supported by the presence of generalized muscle rigidity, jaw trismus, profuse diaphoresis, leukocytosis with neutrophilia, elevated creatine kinase levels, and autonomic instability, all temporally related to the introduction of quetiapine.

Previous studies have shown that 16% of NMS cases begin within 24 hours of antipsychotic exposure, 66% within one week, and nearly all within 30 days [11]. Approximately 82% of patients initially present with either altered mental status or muscle rigidity [12]. Because early signs may be subtle or misattributed, especially in elderly or cognitively impaired individuals, diagnosis is often delayed. Institutionalized patients are particularly vulnerable.

This case posed significant diagnostic and therapeutic challenges. The differential diagnosis with SS was especially relevant given that the patient was receiving both quetiapine and the SNRI duloxetine. This underscores the role of polypharmacy in facilitating pharmacodynamic interactions and adverse drug reactions that require meticulous clinical evaluation.

The clinical overlap between NMS and SS is considerable. The combination of serotonergic agents (e.g., SSRIs, SNRIs) with dopamine antagonists has been implicated in the pathogenesis of NMS. One hypothesis suggests that serotonergic modulation of dopaminergic pathways may increase vulnerability to NMS when combined with antipsychotics. Interestingly, NMS has also been reported in association with serotonergic drugs alone. Case reports have linked duloxetine and venlafaxine to NMS-like presentations, suggesting that dopamine blockade may not be the sole requirement for the syndrome's development [13].

Several other medical conditions may mimic NMS, complicating its diagnosis. These include central nervous system infections (e.g., meningitis, encephalitis), heatstroke, metabolic derangements, toxic encephalopathies, agitated delirium, nonconvulsive status epilepticus, and MH [6]. In this case, an extensive diagnostic workup was performed to rule out these alternative diagnoses.

Despite early recognition and treatment, NMS may not respond adequately to pharmacologic or supportive interventions. Reported mortality rates range from approximately 10% to 20% and may be even higher among elderly patients with significant comorbidities [2,12].

In the present case, several clinical features may have contributed to the fatal outcome. The patient's history of heart failure with reduced ejection fraction likely increased susceptibility to hemodynamic instability, given the narrow margin for fluid management in such individuals. The risk of life-threatening arrhythmias, commonly seen in the setting of severe electrolyte imbalances and autonomic dysregulation, may also have been a contributing factor. Additional poor prognostic indicators included advanced age, multiple comorbidities, functional dependency, and polypharmacy, all of which are known to worsen outcomes in patients with NMS.

The progressive hypernatremia observed in this case reflects a multifactorial disturbance. In addition to limited oral intake and advanced age, hypernatremia may have been further aggravated by dehydration, insensible fluid losses from hyperthermia, and rhabdomyolysis-related fluid shifts. Mild renal dysfunction likely contributed to impaired free water clearance, compounding the electrolyte imbalance. These overlapping factors underscore the complexity of fluid and electrolyte management in patients with NMS, particularly when compounded by frailty and reduced physiological reserve. This case illustrates how systemic complications, such as hypernatremia, can significantly impact outcomes and must be aggressively monitored and managed, even when patient-specific constraints limit therapeutic options.

## Conclusions

NMS continues to pose significant diagnostic and therapeutic challenges, particularly in older adults receiving antipsychotic treatment. Although advanced age has traditionally been associated with poorer outcomes, current evidence does not establish age as an independent risk factor for NMS-related mortality. Instead, the high prevalence of comorbidities and polypharmacy in the elderly may play a more decisive role in determining prognosis, with age potentially acting as a confounding variable. This case emphasizes the need for heightened clinical vigilance in recognizing NMS in vulnerable populations. The presence of fever, rigidity, and altered mental status following changes in antipsychotic therapy should prompt immediate evaluation, especially when accompanied by elevated creatine kinase levels. While early diagnosis and prompt treatment can improve outcomes, clinical deterioration is often influenced by the patient’s overall medical complexity. Risk stratification should therefore account not only for chronological age but also for the burden of comorbidities and functional status. Clinicians must exercise careful judgment when prescribing or modifying antipsychotics, balancing therapeutic goals with the risk of serious, potentially fatal adverse effects such as NMS.
